# Daily Food Insecurity Predicts Lower Positive and Higher Negative Affect: An Ecological Momentary Assessment Study

**DOI:** 10.3389/fnut.2022.790519

**Published:** 2022-03-25

**Authors:** Muzi Na, Nan Dou, Yujie Liao, Sara Jimenez Rincon, Lori A. Francis, Jennifer E. Graham-Engeland, Laura E. Murray-Kolb, Runze Li

**Affiliations:** ^1^Department of Nutritional Sciences, The Pennsylvania State University, University Park, PA, United States; ^2^Department of Statistics, The Pennsylvania State University, University Park, PA, United States; ^3^Department of Biobehavioral Health, The Pennsylvania State University, University Park, PA, United States; ^4^Department of Nutrition Science, Purdue University, West Lafayette, IN, United States

**Keywords:** food insecurity, mood, EMA, season, stress

## Abstract

Food insecurity (FI) is a dynamic phenomenon, and its association with daily affect is unknown. We explored the association between daily FI and affect among low-income adults during a 2-seasonal-month period that covered days both pre- and during the COVID-19 pandemic. A total of 29 healthy low-income adults were recruited during fall in 2019 or 2020, 25 of whom were followed in winter in 2020 or 2021. Daily FI (measured once daily) and affect (measured 5 times daily) were collected over the 2nd−4th week in each month. Time-Varying-Effect-Models were used to estimate the association between daily FI and positive/negative affect (PA/NA). Overall, 902 person-days of daily-level data were collected. Daily FI was associated with lower PA in the 3rd and 4th week of fall and winter and with higher NA in the second half of winter months. Similar patterns of FI-affect relations were found pre- and during COVID-19 in the second half of a given month, while unique patterns of positive affect scores in the 2nd week and negative scores in the 1st week were only observed during COVID days. Our study supports a time-varying association between FI and affect in low-income adults. Future large studies are needed to verify the findings; ultimately, better understanding such associations may help identify, target, and intervene in food insecure adults to prevent adverse mental health outcomes.

## Introduction

Food insecurity is defined as having limited access to adequate food for active, healthy living due to lack of money and other resources, and it disproportionally affects vulnerable populations, including low-income households (1). In 2019, the prevalence of food insecurity in households with income < 185% of the federal poverty line (FPL) was nearly three times higher (27.6%) than the national average (10.5%) (1). During the COVID-19 pandemic, the prevalence of food insecurity increased dramatically to 44% in low-income adults (2). Low-income populations were more susceptible because pandemic lockdown, quarantine, and isolation measures lead to additional financial stress, loss of employment, and incapability to access sufficient food sources in low-income households, with additional anxiety and depression resulting from these stressors (3, 4).

Food insecurity is associated with a wide range of chronic conditions, including diabetes, obesity, cardiovascular diseases, and poor neurocognitive function (5–10). Rather than being static, food insecurity is believed to be a dynamic process that potentially follows a cyclical pattern. Fluctuations in food expenditure and dietary intake over the course of a given month have been observed in low-income adults (11, 12), households (13, 14) and communities (15). Changes in food insecurity by season are also possibly profound in low-income households, where other essential living expenses, such as heating costs in winter, may stress the limited resources for access to healthy foods (16) and create the seasonal “heat-or-eat” dilemma during winter times when heating costs compete with food costs (17).

In addition to its direct impact on diet, food insecurity is associated with increased adverse mental health outcomes (18). The directionality and mechanisms of the food insecurity-mental health relationship, however, are unclear. Prior longitudinal studies suggested a bidirectional association linking food insecurity measures (e.g., food insecurity experience over the past 12 months) and measures of mental health (primarily focused on depression and stress) (19, 20). Affective tendencies, such as positive or negative affect, can be determined by assessing and aggregating emotional states or people's recollections of their feelings, and can be used to predict a variety of health-behaviors and health outcomes (21–24). Affective reactivity to minor stressful events in daily life has received increasingly more attention because it can be an indicator of vulnerability vs. resilience; that is, the way people react emotionally to daily stressors may be indicative of long-term health and wellbeing (25, 26). Among the low-income population who are at higher risk of food insecurity, the ways in which daily food insecurity relates to everyday affect, and specifically, how the cyclical nature of food insecurity is associated with emotional wellbeing at different times in a given month, and in different times of the year, has not been extensively explored.

Ecological momentary assessment (EMA), using repeated collection of real-time data in subjects' natural living environments, is a useful tool for collecting dynamic data. Compared to the commonly used retrospective reporting, the concurrent behaviors and experience captured by EMA are believed to minimize recall bias and maximize ecological validity (27, 28). EMA has been widely used to assess human dietary intake (29) and affect (30). Using the EMA design on smartphones, we piloted a study designed to track daily food insecurity, dietary intake, and positive and negative affect in everyday life. Our aims were to study: (1) how food insecurity status within a given month in two seasons was associated with affect in predominantly rural, low-income adults on a daily basis; and (2) how the COVID pandemic may modify the association between daily food insecurity and affect. We hypothesized that food insecurity is associated with increased negative affect and decreased positive affect, and that the associations would be more evident in the 4th week than the 2nd week, in the winter than the fall season, and during the pandemic compared to pre-pandemic.

## Materials and Methods

### Subjects and Settings

The Food ‘N Mood study is a pilot study conducted in central Pennsylvania between September 2019 and March 2021. Participants were recruited *via* study flyers from selected communities and locations serving low-income populations, such as Women, Infants and Children (WIC) offices, county assistance offices, food banks, food pantries, and Head-Start childcare services. The inclusion criteria were healthy adults aged 20–50 years and household income below the 185% of the Federal Poverty Line (FPL). The exclusion criteria were adults who were Non-English-speaking, had diagnosed physical, mental or emotional disabilities, who had disabled family members in the household, and those who reported taking medications known to affect heart rate. Women who were pregnant or menopausal were also excluded. Data collection occurred at participants' homes over two 3-week-long waves (between the 2nd and 4th week), covering one fall month (September, October, or November) in 2019 or 2020, and one winter month (February or March) in 2020 or 2021. Data on food insecurity status and affective wellbeing were collected through an EMA framework on provided smartphones. Participants responded to 8 or 9 survey modules on a daily basis, including a morning survey (*n* = 1), notified surveys (*n* = 5), an evening survey (*n* = 1), and food records (Sunday, Monday, and Tuesday of each survey week). A written consent has been obtained from participants enrolled during in-person procedures. During the COVID-19 pandemic, a verbal consent form was filled out during virtual recruitment procedures and an electronic consent form was sent to participants for their records. Participants were provided with up to $140 cash compensation if they completed data collection in both fall and winter season. The study protocol was approved by the National Center for Advancing Translational Sciences (NCATS) and The Pennsylvania State University Institutional Review Board, University Park, Pennsylvania.

### Daily Food Insecurity

Individual level food insecurity status was assessed using an adapted 6-item U.S. Adult Food Security Survey Module given our research interest in adult food insecurity and participants' affect (31). Each day, participants were asked whether or not they had any experience of food insecurity in the past 24 h. The experiences included: “worrying about food running out”, “could not afford to eat balanced meals”, “cutting meal size or skipping a meal because there wasn't enough money for food”, “eating less because there wasn't enough money for food”, “was hungry but did not eat because there wasn't enough money for food” and “did not eat for a whole day because there wasn't enough money for food”. In our study, the mean Cronbach's alpha for the continuous daily food insecurity score, calculated from the sum of the six questions, was 0.37. Given the low alpha value, we chose to generate a binary food insecurity status variable to indicate whether or not the adults experienced any food insecure situations (coded as “1” if participants experienced one or more of these food insecure situations; or “0” if participants did not affirm any of the food insecure experiences). As a set of sensitivity analyses, we applied the continuous food insecurity score in the analysis; results are presented in Supplementary Material.

### Daily Affect

Momentary mood/affective states were assessed *via* an 11-item questionnaire, using items drawn from the Profile of Mood States (32), Positive and Negative Affect Schedule (33), and the modified Differential Emotions Scale (34) to incorporate items reflecting both activated and deactivated positive affect and negative affect. Positive affect was assessed with five items (happy, hopeful, excited, calm, and proud), and negative affect was assessed with six items (tense/anxious, lonely, annoyed, embarrassed, depressed, and tired). The smartphone randomly beeped around five anchor time points with survey reminders, and the participants were asked to rate their mood at the moment on a slider scale that ranged from “not at all” to “extremely” (and which equated to a 0–10 scale). These surveys were considered complete if all of the 11 items were rated. Daily positive and negative affect scores were calculated by computing means of the completed ratings within the same survey day of each participant. Following methods described by Cranford et al. (35), we calculated the reliability for within-person change for positive and negative affect. Both positive and negative affect showed good reliability (R_c_ = 0.98 for both affect measures).

### Demographic and Socioeconomic Characteristics

A background survey was administered at enrollment to collect demographic, health and socioeconomic characteristics of participants, including age, gender, race and ethnicity, relationship status, weight, height, education, employment status, household size, number of children under 18 years in the household, total annual household income, and enrollment in food assistance programs. In addition to the main measure of daily food insecurity, we also measured household food insecurity over the past 12 month as part of the socioeconomic module, using the U.S. Adult Food Security Survey Module (31). Body mass index (BMI) was calculated as self-reported weight (kg)/height^2^ (m^2^). Poverty status was categorized per the Department of Health and Human Services' definition that considers the gross household income (<130% FPL or ≥130 and <185% FPL) and household size (36). To be able to examine whether findings were affected by the COVID-19 pandemic, we created a dummy code to differentiate between “pre-COVID-19 period” and “during COVID-19 period” based on whether data were collected before or after March 1, 2020.

### Statistical Analysis

Time Varying Effect Models (TVEMs) (37, 38) were applied to estimate the association between daily food insecurity status and affect as a function of time. The TVEMs have no constraints on the shapes of coefficients and are free of model misspecification (39). TVEMs have been widely applied for estimating time-varying effects in human behavioral and health research (39–42). Because the coefficient functions for intercept terms were approximately constant (that is the population mean of positive and negative affect did not vary over the course of the month when the explanatory variables are set to be 0), we considered the TVEM model with fixed intercepts and with a coefficient function of daily food insecurity and time using the polynomial spline-based method (43–45). We set the degree of splines to be 3 and the number of knots to be 2 in our final model, which had the lowest Akaike Information Criterion (AIC) in comparison to alternative models. The “geeglm” function with correlation structure “ar1” in R was used to account for intrapersonal correlation of repeated measures in the affect measures. The specific model is described as the following:


$$\begin{array}{l} y_{ij}=\;\beta_{0}+\;\beta_{1}\left(t_{ij}\right)*\;FoodInsecurity_{ij}+\;\gamma^{T}covariates_{i}+\;\epsilon_{ij} \end{array}$$

Where *y*_*ij*_ is the expected daily affect (positive or negative) for subject *i* on day *j*; β_0_ is the fixed intercept (non-time varying) of daily affect; β_1_(*t*_*ij*_) denotes the time-varying association between daily food insecurity and daily affect for subject *i* on day *j*. The 95% pointwise confidence interval for the adjusted β_1_(*t*_*ij*_) was estimated across the 21 study days within a month to determine statistical significance. $\gamma ={\left(\gamma_{1},\;\ldots ,\;\gamma_{4}\right)}^{T}$ is the vector of parameters for covariates being adjusted in the model, including gender, race/ethnicity, employment, poverty status, and whether or not data collection occurred during COVID-19. These covariates were considered because of their known relationship to food insecurity and emotional health (46–48). γ is considered constant for different individuals over time. ε_*ij*_ is the continuous error term that was not assumed to follow a normal distribution as suggested by previous work (41).

To examine the potential role of COVID-19 with the food-insecurity-affect associations, the observations of food insecurity and affect were stratified by data collection pre- or during the COVID-19 period, controlling for gender, race/ethnicity, employment, poverty status, and whether data were collected in fall or winter months in otherwise similar TVEMs.

## Results

Overall, 29 participants were enrolled in the study. A sample of 28 participants were recruited in fall 2019/2020, of whom 25 participants were followed up in winter 2020/2021; one additional participant was recruited in winter 2020/2021. A total of 902 person-days (response rate = 78.1%) or an average of 30.1 days per participant of information on food insecurity and affect were collected. Table 1 presents the baseline demographic and socioeconomic characteristics of the 29 participants. The mean (SD) age of participants was 36.3 (7.1) years. The majority of adults enrolled were women (86.2%), White or Caucasian (69.0%), employed (72.4%), and had college or higher degrees (82.8%). Sixteen participants who were employed at baseline (*n* = 21) provided information about their payment schedule in the survey month, including two who were paid weekly, 8 who were paid bi-monthly, and 6 who were paid monthly. About half of the participants were married or living with partners (48.3%). Sixteen (55.2%) participants lived below the 130% FPL and thirteen (44.8%) lived between 130 and 185% FPL.

**Table 1:** Characteristics of the low-income participants (<185% of FPL) at enrollment^a, b^.

	**Mean (SD) or *n* (%)**
**Age**	36.3 (7.1)
**Female**	25 (86.2%)
**Race/ethnicity**	
White or Caucasian	20 (69.0%)
Black or African American, Asian, and Other	9 (31.0%)
**Education**	
Less than high school, high school/GED, OR some college	5 (17.2%)
College and above	24 (82.8%)
**Employment**	
Employed	21 (72.4%)
Unemployed	8 (27.6%)
**Payment schedule among the employed (*****N*** **=** **21)**	
Monthly, by the end of 1st week	1 (4.8%)
Monthly, by the end of 2nd week	1 (4.8%)
Monthly, on the last day of month	4 (19.0%)
Bi-monthly, by the end of 1st week and 3rd week	4 (19.0%)
Bi-monthly, by the end of 2nd week and 4th week	4 (19.0%)
Weekly	2 (9.5%)
Missing	5 (23.9%)
**Marital status**	
Married or living with partner	14 (48.3%)
Single, divorced, widowed	15 (51.7%)
**Household size**	4.0 (1.7)
**Number of children under 18 years in the household**	2.2 (1.7)
**Poverty status**	
≥130% FPL	13 (44.8%)
<130% FPL	16 (55.2%)
**Food assistance programs, SNAP/food stamps in the past 12 months**	8 (27.6%)
**BMI, kg/m**^**2**^ **(*****N*** **=** **22)**	27.8 (8.4)

*^a^BMI, body mass index; FPL, federal poverty line; SNAP, Supplemental Nutrition Assistance Program*.

*^b^Mean (SD) were reported for continuous variables including age, household size, number of children under 18 years in the household, BMI, and depressive symptoms. N (%) were reported for categorical variables including gender, race/ethnicity, education, employment, payment schedule, marital status, household income, poverty status, and participation in food assistance programs*.

**Figure 1:**
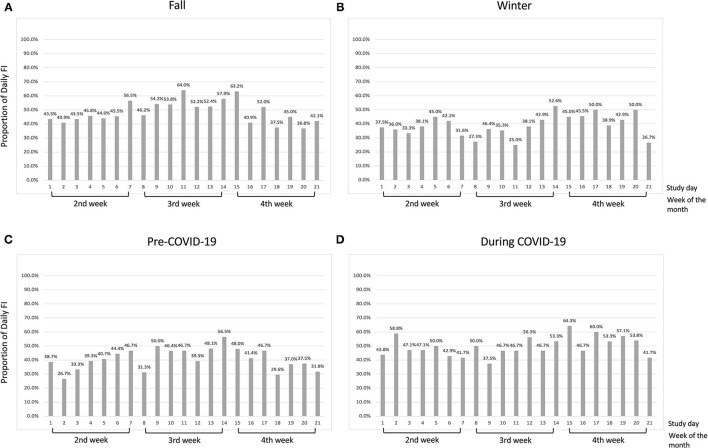
Proportion of daily food insecurity by study day in low-income participants (<185% of FPL) in **(A)** the fall months; **(B)** the winter months; **(C)** pre-COVID-19 period; and **(D)** during COVID-19 period.

The proportion of daily food insecurity reported by participants is presented in Figure 1. In the fall months, the proportion of participants with any daily food insecurity experience ranged from 36.8 to 64.0%. There were 8 days in which over half of the participants affirmed that at least one food insecurity situation occurred (Figure 1A). In the winter months, the proportion range was 25.0–52.6% and there were 3 days in which more than half of the participants reported experiencing at least one food insecurity situation (Figure 1B). We observed a higher proportion of daily food insecurity reported during the COVID pandemic than the pre-pandemic period. The daily proportion range was 26.7–56.5% and 37.5–64.3%, respectively, for the pre- and during COVID periods. The number of days during which half or more participants had any food insecurity situation increased from 2 days in a given month pre-COVID to 10 days in a given month during the COVID pandemic (Figures 1C,D).

**Figure 2:**
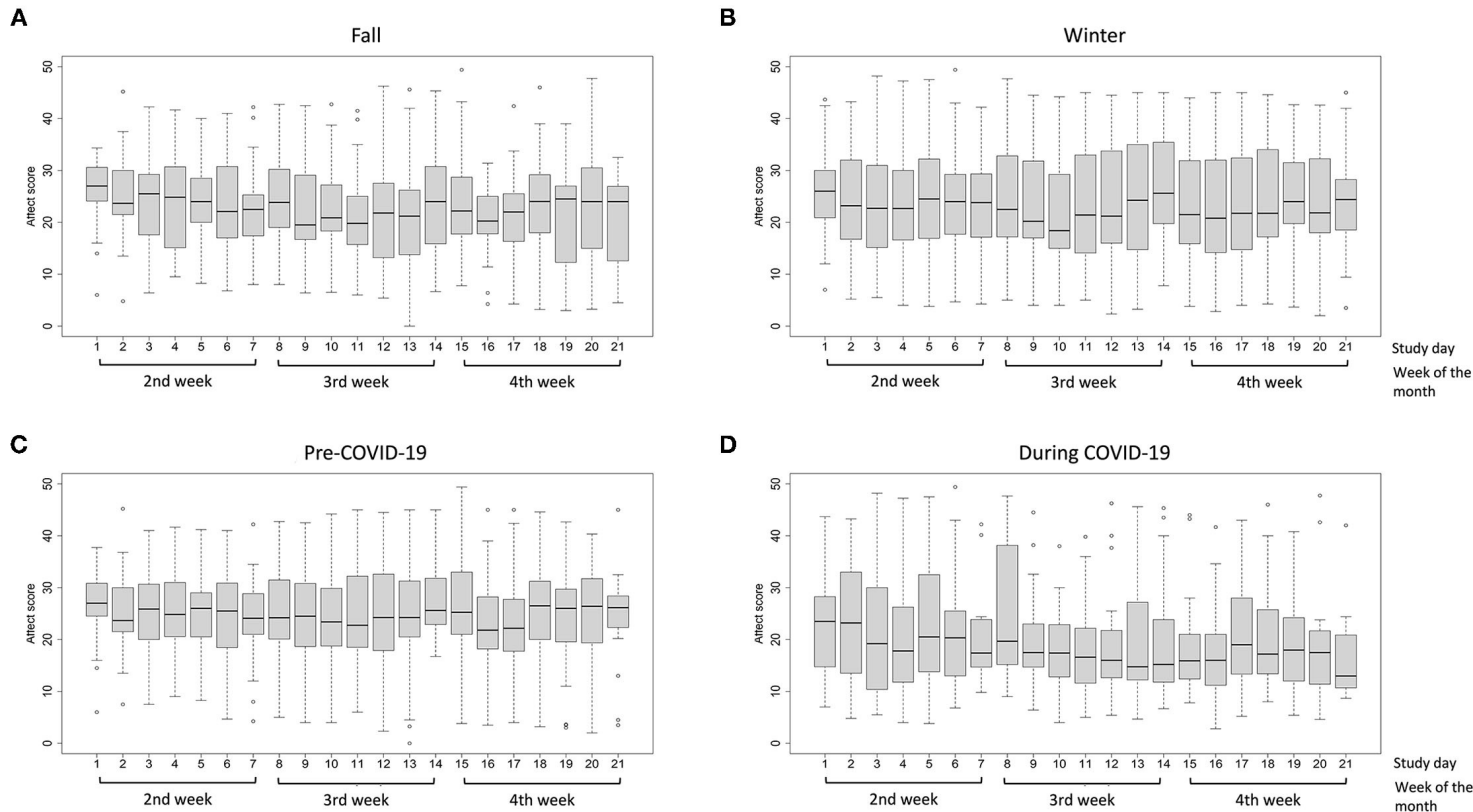
Distribution of positive affect scores in low-income participants (<185% of FPL) by study day **(A)** the fall months; **(B)** the winter months; **(C)** pre-COVID-19 period; and **(D)** during COVID-19 period. The boxplot indicates the minimum score (bottom line), 25th percentile (lower bound), median (middle bar), 75th percentile (upper bound), and maximum score (top line) of positive or negative affect on each study day. The circles below and above the boxplots indicate outliers.

**Figure 3:**
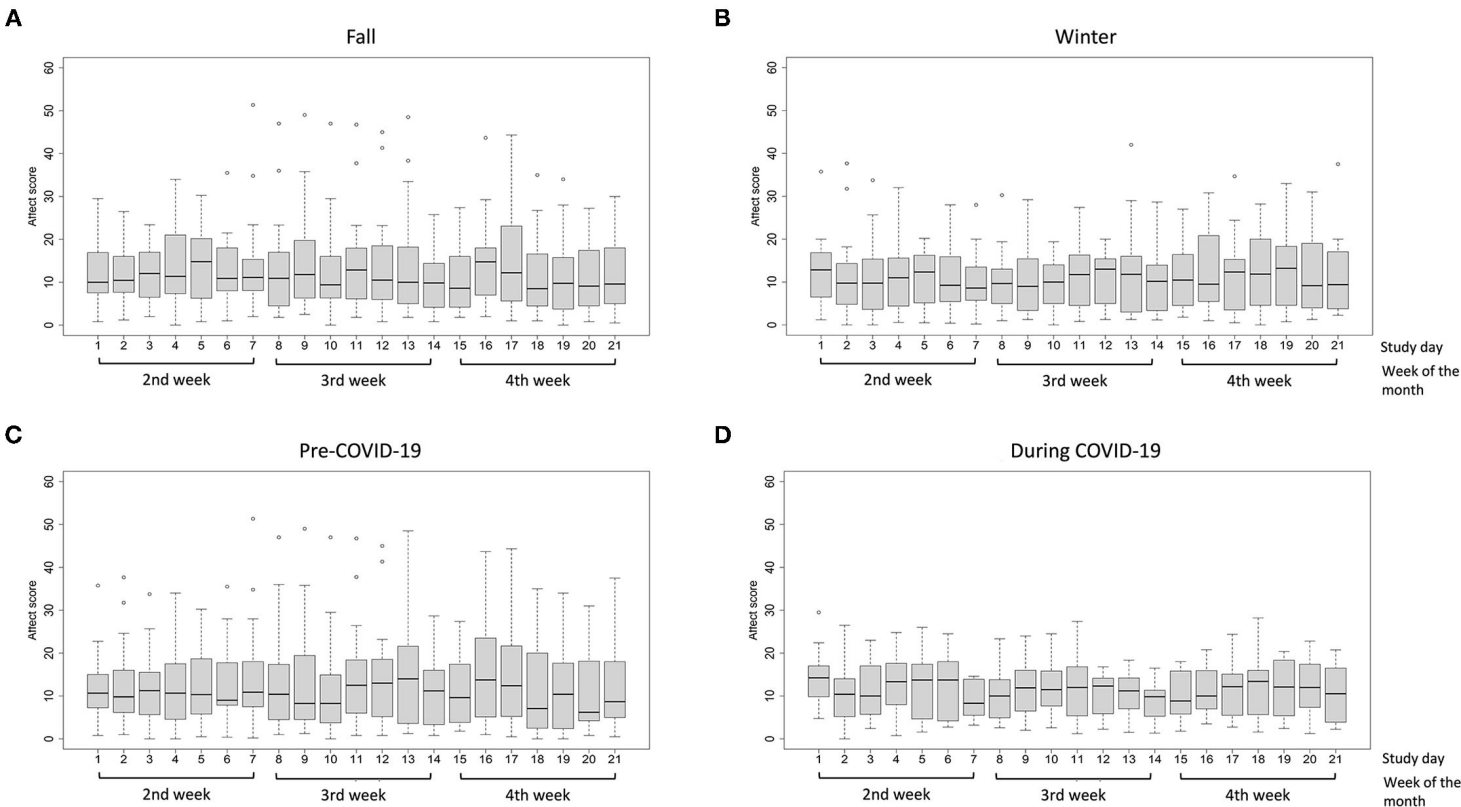
Distribution of negative affect scores in low-income participants (<185% of FPL) by study day **(A)** the fall months; **(B)** the winter months; **(C)** pre-COVID-19 period; and **(D)** during COVID-19 period. The boxplot indicates the minimum score (bottom line), 25th percentile (lower bound), median (middle bar), 75th percentile (upper bound), and maximum score (top line) of positive or negative affect on each study day. The circles below and above the boxplots indicate outliers.

As depicted in Figures 2, 3, daily positive and negative affect showed wide interpersonal variation each day. For positive affect, the median score was 23.0 in both fall and winter months (Figures 2A,B). The median score was 24.7 in the pre-COVID period and 17.3 during COVID (Figures 2C,D). For negative affect (Figure 3), the median for fall (Figure 3A), winter (Figure 3B), pre-COVID (Figure 3C) and COVID periods (Figure 3D) was 10.7, 10.6, 10.0, and 11.5, respectively. Out of the maximum reporting of 5 times per day, the average daily frequency of reporting was 3.4 for both positive and negative affect.

**Figure 4:**
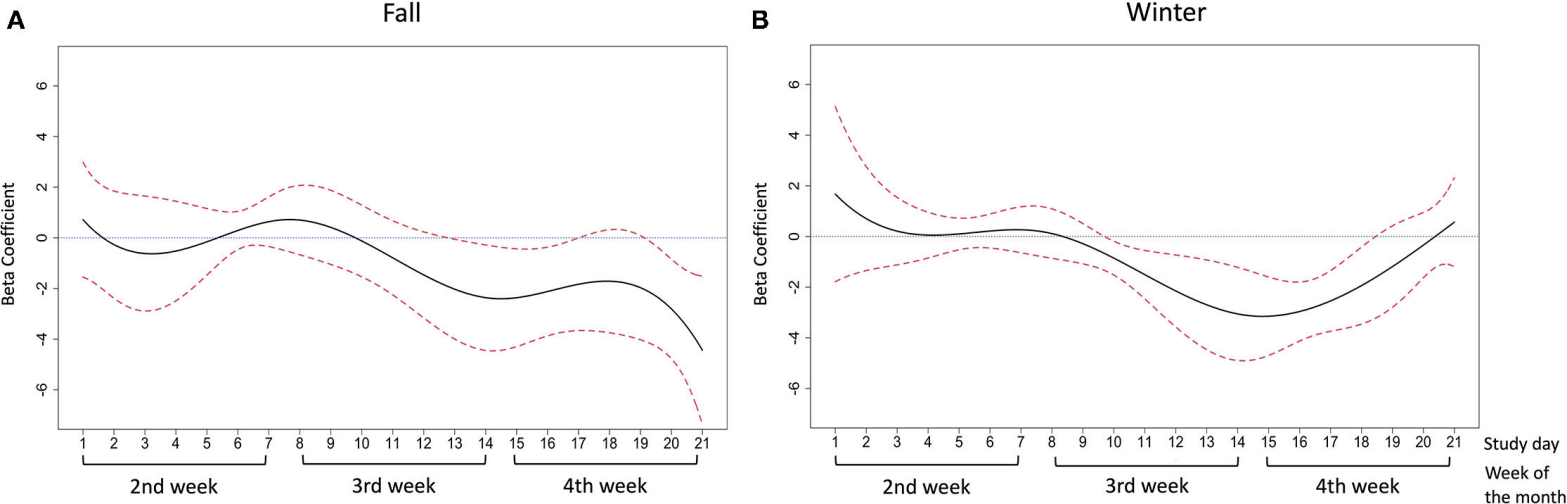
The association between daily food insecurity status and positive affect in **(A)** the fall months; **(B)** the winter months. The time-varying effect models were adjusted for gender, race/ethnicity, employment, poverty status, and data collection pre- or during covid-19. The solid line in black represents the estimated coefficients between daily food insecurity status and positive affect. The dashed lines in red are the pointwise 95% confidence intervals. The dotted blue line at zero represents null association between daily food insecurity and affective wellbeing.

**Figure 5:**
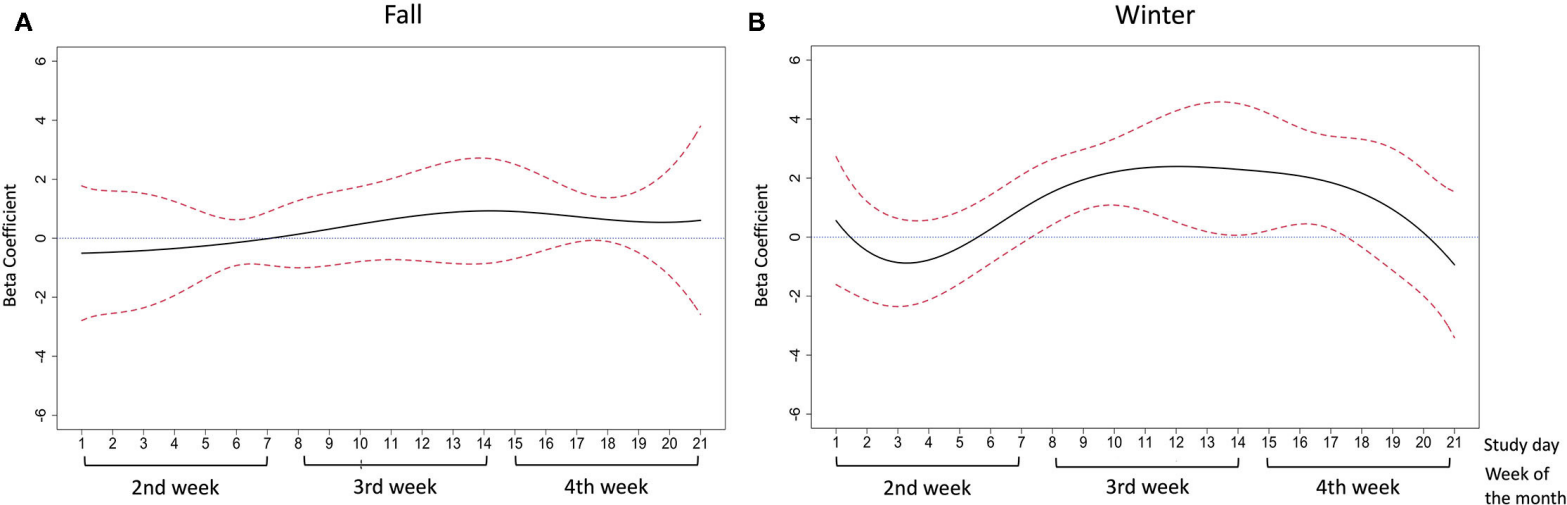
The association between daily food insecurity status and negative affect in **(A)** fall months; **(B)** winter months. The solid line in black represents the estimated coefficients between daily food insecurity status and negative affect. The dashed lines in red are the pointwise 95% confidence intervals. The dotted blue line at zero represents null association between daily food insecurity and affective wellbeing.

The adjusted relationships were controlled for gender, race/ethnicity, employment, poverty status, and whether or not data assessment occurred during the COVID-19 pandemic. In the fall months, daily food insecurity was associated with lower positive affect scores between study days 10–21 and significant associations were seen between study days 13–17 (the end of the 3rd week and the beginning of the 4th week, beta-coefficient ranged from −2.37 to −1.83) and study days 20–21 (the end of the 4th week, beta-coefficient was −2.81 and −4.44, respectively) (Figure 4A). Experiencing daily food insecurity was significantly associated with lower positive affect scores and the coefficients decreased between study day 13–15, and slightly increased in study day 16–17. In the winter months, a similar negative association was observed for positive affect between study days 9–20 and the significant association was observed during study days 10–18 (most of the 3rd week and 4th week of the month, beta-coefficient ranged from −3.15 to −0.86) (Figure 4B). The coefficients decreased between study day 10–15 and slightly increased between study day 16–18. Contrary to the results observed for positive affect, there was no association between daily food insecurity and negative affect in the fall months (Figure 5A). Daily food insecurity was significantly associated with higher negative affect scores in the winter months during study days 8–17 (3rd and most of the 4th week of the month, beta-coefficient ranged from 1.53 to 2.39), where the coefficients increased first in study day 8–12, and then decreased in study day 13–on study day 17 (Figure 5B).

**Figure 6:**
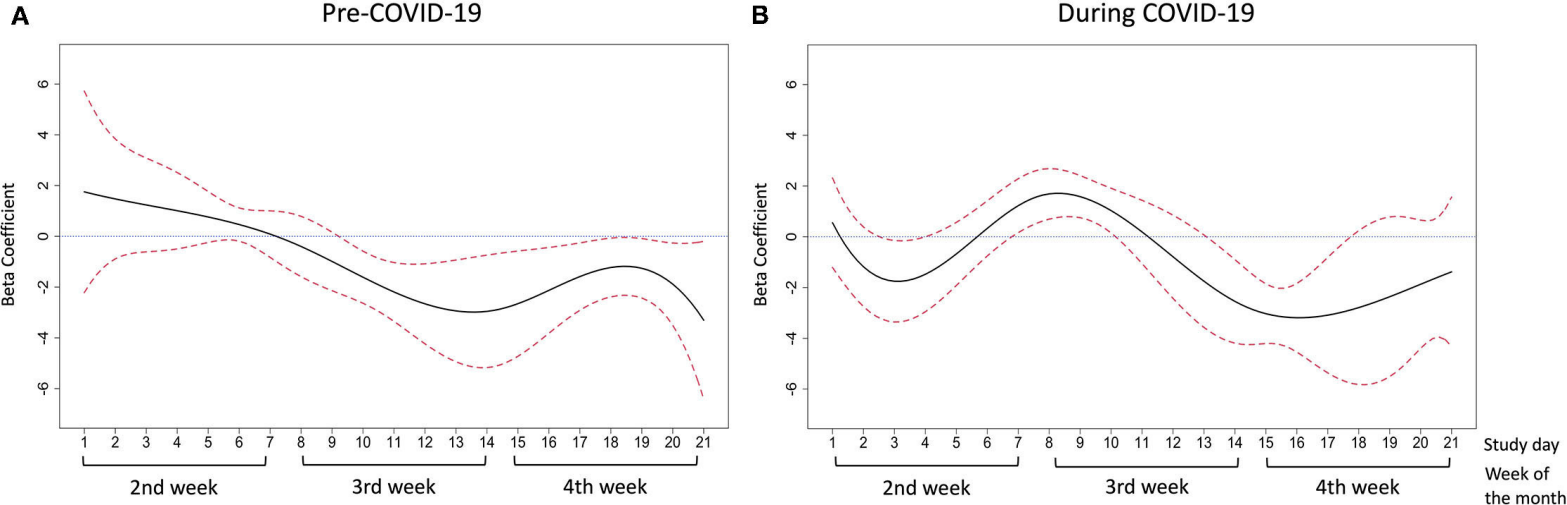
The association between daily food insecurity status and positive affect **(A)** in pre-COVID-19 days; **(B)** during COVID-19 days. The time-varying effect models were adjusted for gender, race/ethnicity, employment, poverty status, and season. The solid line in black represents the estimated coefficients between daily food insecurity status and positive affect. The dashed lines in red are the pointwise 95% confidence intervals. The dotted blue line at zero represents null association between daily food insecurity and affective wellbeing.

**Figure 7:**
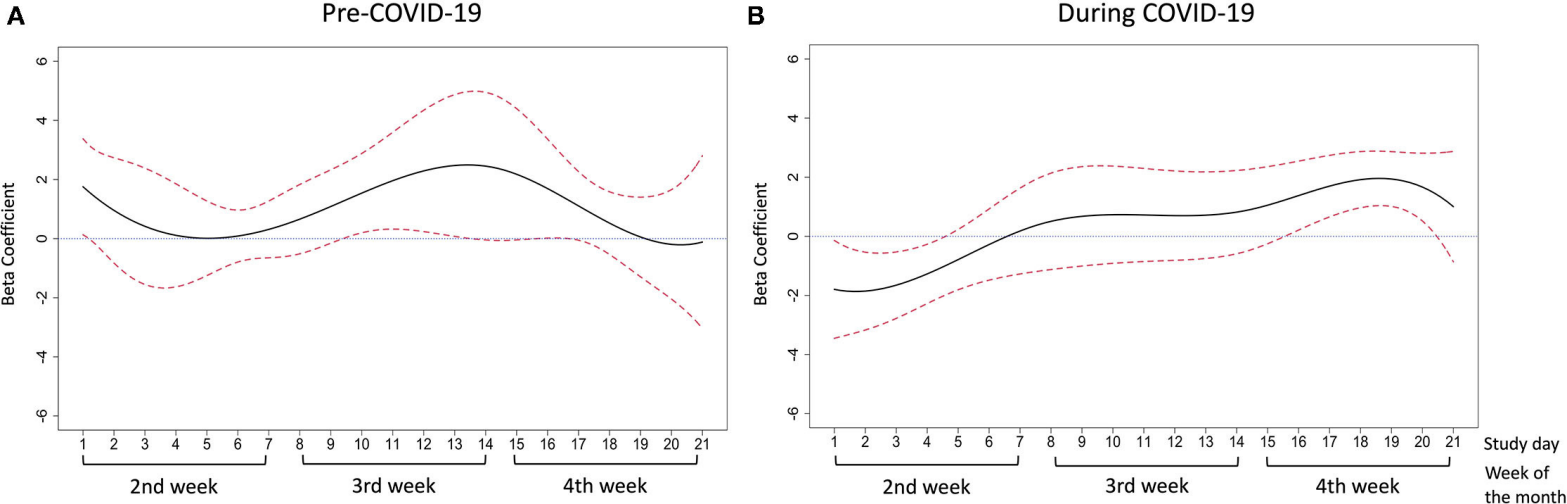
The association between daily food insecurity status and negative affect **(A)** in pre-COVID-19 days; **(B)** during COVID-19 days. The time-varying effect models were adjusted for gender, race/ethnicity, employment, poverty status, and season. The solid line in black represents the estimated coefficients between daily food insecurity status and negative affect. The dashed lines in red are the pointwise 95% confidence intervals. The dotted blue line at zero represents null association between daily food insecurity and affective wellbeing.

The adjusted associations between daily food insecurity status and affect pre- and during the COVID-19 pandemic are presented by study day in Figure 6 for positive affect and Figure 7 for negative affect. The detailed estimates are presented in Supplementary Tables 3, 4.

Prior to the COVID-19 pandemic, daily food insecurity was significantly associated with lower positive affect scores between study days 10–21 (the middle of the 3rd week to the end of the 4th week of the month, beta-coefficient ranged from −3.30 to −1.23) (Figure 6A). A decreasing trend of coefficients was observed between study day 10–14. Between study day 15–21, the coefficients increased first then deceased. During the COVID-19 pandemic, daily food insecurity was significantly associated with lower positive affect scores between study day 3–4 (the middle of the 2nd week of the month, beta-coefficient was −1.78 and −1.48, respectively) and study day 14–17 (the end of the 3rd week and the 4th week of the month, beta-coefficient ranged from −3.19 to −2.55) (Figure 6B). From study days 7–10 (the last day of the 1st week and the beginning of the 2nd week), experiencing food insecurity was associated with increased positive affect (beta-coefficients ranged between 1.03 and 1.69).

For negative affect in pre-pandemic days, daily food insecurity was significantly associated with higher negative affect scores from study day 10–13 and to study day 16 (most of the 3rd week of the month, beta-coefficient ranged 1.54–2.48) (Figure 7A). The coefficient increased between study day 5–13, and showed a decreasing trend afterwards until study day 21 (Figure 7A). During the pandemic, daily food insecurity was significantly associated with higher negative affect from study day 16–20 (most of the 4th week of the month, beta-coefficient ranged 1.38–1.94) (Figure 7B). However, during study days 1–4 (the first half of the 2nd week in the month), we also observed lower negative affect scores associated with daily food insecurity (beta-coefficient ranged −1.86 to −1.27; Figure 7B). Overall, the beta-coefficient for negative affect generally increased from day 2–20.

Using the continuous daily food insecurity, instead of the dichotomous daily food insecurity status, we found similar and robust results from similarly constructed TVEMs (Supplementary Figures 5, 6).

## Discussion

In this pilot study, we measured daily food insecurity and affect among 29 low-income adults, 25 of whom were tracked for two 21-day periods over two seasons. We identified a time-varying association of daily food insecurity with daily affect within a given month. Regardless of season and whether the data were collected pre- or during the COVID-19 pandemic, we have observed a generally consistent association between daily food insecurity and lower positive affect and higher negative affect in the second half of a given month. Using intensive longitudinal data and an EMA design, our study was the first to track day-to-day food insecurity stressors and affect and demonstrate how low-income adults' affect fluctuates with food insecurity experiences in daily life. Given that prior research has focused more on chronic food insecurity in relation to mental health outcomes, findings from this study are novel and may suggest that psychological vulnerability to minor, possibly transient, stressors related to food insecurity are time-varying within a given month and across different times of the year.

Daily negative and positive affect were associated with daily food insecurity in certain weeks of the months. Both positive and negative affective reactivity to same-day stressful events, along with one's affective variability over time, seem to have independent health implications (49–53). Our study findings suggest that food insecurity may affect health in ways outside of diet-dependent pathways, with affect perhaps serving as an important mechanism in what has been termed the “cycle of food insecurity and chronic disease” model (54). However, caution is needed when interpreting our data, because the directionality of the associations cannot be confirmed and stress-related biomarkers were not incorporated in the present work. More research is needed to test the hypothesis that food insecurity affects physical health and to determine mechanisms of this potential relationship.

Although we did not see an increasing pattern of reported daily food insecurity over week (Figure 1), we did observe that the food insecurity-affect relationship became stronger in the expected direction in the latter half of the week (Figures 4–7). This phenomenon may be partially explained by the anticipated cycle of arrival and depletion of income/ benefits within a given month and the coping strategies available to “make ends meet”. Low-income adults may face higher economic strain near the end of the month if they get most of their income at the beginning or on the last day of the month. In addition, SNAP benefits in Pennsylvania are distributed to eligible low-income households within the first 10 days of each month (55). SNAP benefits are usually spent quickly within the first few days after receipt (13, 14), leaving the rest of the month less likely to be supported by SNAP benefits. In exploratory analyses to test how payment cycle may modify the relationship, we grouped people by whether or not they received money in the 4th week and observed decreased positive affect and increased negative affect in the 4th week among people who did not receive any payment in that week (*n* = 14). Similarly, for people who did receive payment in the 4th week (*n* = 8), similar decreased positive and increased negative affect scores were observed but were shifted to 1 week earlier (data not shown), suggesting the possibility that such individuals may have exhausted their resources by the week before the next payment. Because of the small sample size and missing data in payment cycle information, future studies are warranted to explore the role of payment cycle in the food insecurity and affect relationship.

In addition, low-income adults may have time-varying stress reactivity in response to food insecurity due to limited coping strategies in latter half of the months. Responsiveness to financial stress, characterized by both primary coping strategies (i.e., stressful situation management through problem solving, emotional expression, and emotion regulation) and secondary coping strategies (i.e., stressful situation adaptation through active acceptance, cognitive restructuring, distraction, and positive thinking) is related to how well low-income adults adapt to stress (56). Specifically, strategies for food budgeting that help stretch available food dollars, referred to as food management skills (57), were found to mitigate the impact of food insecurity in low-income adults and children (58–61). Coping with food insecurity using food management skills could be increasingly challenging when food dollars become limited at the end of the month. While low-income people may be able to secure food, they may have to sacrifice other critical resources in doing so, such as cost-related medication needs (62), in ways that may directly or indirectly influence their mental wellbeing. Findings are consistent with the possibility that food insecurity contributes to changes in affect. However, causality cannot be determined from the present research. It is possible that additional factors may have contributed to the observed time-varying relationships. For example, other stressors may be associated with both food insecurity and affect.

The COVID-19 pandemic appears to have modified the association between daily food insecurity and affect. While the associations in the pre-pandemic period largely mimicked what we saw in the fall and winter, some of the ups and downs in affect were in the unexpected direction during the COVID-19 pandemic. We do not fully understand why participants who reported having daily food insecurity experience appeared to feel more “hopeful” than their food-secure peers during the COVID-19 pandemic. This may be due to the additional COVID-19 relief benefits that they were eligible to receive or that they had already received. Unfortunately, we do not have the data necessary to verify this hypothesis. However, experiencing food insecurity was associated with increasingly worst affect in the 3rd and 4th week of the months, confirming the second half of the months as the most vulnerable time regardless of whether it was pre- or during the pandemic period.

Our study has several limitations. The small sample size prevented us from conducting an analysis by food security level or by subject characteristics. Participants tended to skip a few notified surveys within a day; however, the majority of participants (25/30 or 83.3%) completed at least 1 affect survey on each study day and the mean daily frequency was more than three times. The pandemic may have influenced patterns in the association between food insecurity and affect, although the pandemic indicator included in all of our adjusted models was not statistically significant. The findings by COVID-19 status revealed some different patterns when comparing the pre- and during the pandemic periods. The residual confounding may not be fully controlled for by adding whether or not data were collected before or after March 2020. For example, both food access and affect may have been impacted by the pandemic because of the change in self or family members' health conditions, employment status, income, and participation in food assistance programs and COVID-related financial assistance programs during the study period; however, our study did not collect these data, so we were not able to adjust for these factors. We did not assess every type of daily stressor that may contribute to affect variability. There may be unmeasured variables that account for the observed association. BMI data were missing in 9 (31%) of the participants, but sensitivity analysis with further adjustment of BMI revealed similar results (data not shown). Because of the potential residual confounding, our findings in this small pilot study should be interpreted with caution. Future observational studies with large sample size and detailed measures of daily stressors and behaviors are warranted to further explore the time-varying FI-affect associations. More than 80% of participants had a college degree or higher, which was much higher than the average in Pennsylvania (63). Although we followed the standard protocol strictly to screen for eligible low-income adults, potential for overreporting of educational attainment and/or underreporting of household income was possible. Future studies are needed to investigate the association between food insecurity and affect in low-income populations of lower educational attainment. The majority of the participants (86.7%) were female. Compared to males, females endure a higher burden of affect and stress related diseases (64). Our research findings, therefore, may not represent low-income adults as a whole. Lastly, our findings regarding seasonal differences may not be generalizable to other regions.

There are several strengths of the study to be recognized. Using an EMA model on smartphones, our study applied an intensive longitudinal design that assessed daily food insecurity status and multiple affective measures within a survey day, thus reducing measurement error and recall bias. Utilizing the USDA 6-item food insecurity module to assess the daily food insecure status was novel. Although further validation of the daily food insecurity measure is needed, data collected from the same pilot study suggested that daily food insecurity is associated with poor overall dietary quality (65), which provided some external validity of using the current daily FI indicator. The rigorous time-varying statistical models used in food insecurity research are a new extension and a strength. Albeit in a small sample, the overall trends of time-varying affect change in relation to daily food insecurity provide the first empirical evidence of the interplay between food insecurity and affect, and their potential association with the food insecurity cycle.

## Conclusion

Food insecurity is prevalent among low-income adults and is associated with their day-to-day affective wellbeing. This pilot study provides empirical evidence of a time-varying association in the latter half of a month between daily food insecurity and positive mood in fall and winter months and between daily food insecurity and negative moods in winter months only. It also suggests that the COVID-19 pandemic may have influenced the associations between daily food insecurity and affect across the month. Larger observational studies are needed to further verify these preliminary findings. Such research holds promise to better identify at-risk populations and may eventually lead to more timely prevention and intervention efforts to minimize food insecurity and promote mental health in low-income populations.

## Data Availability Statement

The raw data supporting the conclusions of this article will be made available by the authors, without undue reservation.

## Ethics Statement

The study was conducted according to the guidelines of the Declaration of Helsinki, and approved by the National Center for Advancing Translational Sciences (NCATS) and the Institutional Review Board of the Pennsylvania State University, University Park, Pennsylvania (Protocol code: STUDY00010715, Date of approval: 12/02/2018). The patients/participants provided their written informed consent to participate in this study.

## Author Contributions

MN, LF, JG-E, LM-K, and RL: conceptualization and methodology. MN: funding acquisition, project administration, and writing—original draft preparation. MN and ND: data collection and curation. ND, YL, and RL: formal analysis. MN, ND, YL, SJR, LF, JG-E, LM-K, and RL: writing—review and editing. All authors contributed to the article and approved the submitted version.

## Funding

This project described was supported by the National Center for Advancing Translational Sciences, National Institutes of Health, through Grant UL1TR002014. The content is solely the responsibility of the authors and does not necessarily represent the official views of the NIH. MN's effort was also supported by the Broadhurst Career Development Professorship for the Study of Health Promotion and Disease Prevention, Penn State.

## Conflict of Interest

The authors declare that the research was conducted in the absence of any commercial or financial relationships that could be construed as a potential conflict of interest.

## Publisher's Note

All claims expressed in this article are solely those of the authors and do not necessarily represent those of their affiliated organizations, or those of the publisher, the editors and the reviewers. Any product that may be evaluated in this article, or claim that may be made by its manufacturer, is not guaranteed or endorsed by the publisher.
