# Supplementary material for: Daily Food Insecurity Predicts Lower Positive and Higher Negative Affect: An Ecological Momentary Assessment Study
Source: Front Nutr. 2022 Mar 25;9:790519. doi: 10.3389/fnut.2022.790519 (PMC8990300; doi:10.3389/fnut.2022.790519)
Supplement: Supplementary file 1 [file Data_Sheet_1.docx]

Supplementary Material

**Supplemental Table 1.** The estimates (95%CIs) from the time-varying effect model for positive affect score in the fall and winter months using binary food insecurity status ^1,2^

|  | **Fall** | | **Winter** | |
| --- | --- | --- | --- | --- |
|  | **b** | **95% CI** | **b** | **95% CI** |
| **Food insecurity status per study day** | | | | |
| 1 | 0.71 | (-1.55, 2.98) | 1.67 | (-1.78, 5.13) |
| 2 | -0.27 | (-2.39, 1.85) | 0.70 | (-1.35, 2.75) |
| 3 | -0.62 | (-2.89, 1.65) | 0.21 | (-1.12, 1.54) |
| 4 | -0.52 | (-2.49, 1.44) | 0.05 | (-0.85, 0.96) |
| 5 | -0.16 | (-1.47, 1.16) | 0.10 | (-0.52, 0.72) |
| 6 | 0.29 | (-0.46, 1.04) | 0.21 | (-0.46, 0.89) |
| 7 | 0.64 | (-0.34, 1.62) | 0.26 | (-0.64, 1.17) |
| 8 | 0.70 | (-0.67, 2.07) | 0.11 | (-0.86, 1.09) |
| 9 | 0.41 | (-1.06, 1.88) | -0.28 | (-1.08, 0.51) |
| 10 | -0.12 | (-1.54, 1.29) | **-0.86** | **(-1.52, -0.19)** |
| 11 | -0.79 | (-2.25, 0.67) | **-1.51** | **(-2.46, -0.56)** |
| 12 | -1.46 | (-3.16, 0.23) | **-2.15** | **(-3.57, -0.73)** |
| 13 | **-2.03** | **(-4.00, -0.06)** | **-2.70** | **(-4.47, -0.93)** |
| 14 | **-2.36** | **(-4.45, -0.28)** | **-3.06** | **(-4.90, -1.22)** |
| 15 | **-2.37** | **(-4.30, -0.43)** | **-3.15** | **(-4.70, -1.60)** |
| 16 | **-2.12** | **(-3.87, -0.37)** | **-2.96** | **(-4.13, -1.80)** |
| 17 | **-1.83** | **(-3.66, -0.01)** | **-2.55** | **(-3.74, -1.35)** |
| 18 | -1.71 | (-3.75, 0.32) | **-1.94** | **(-3.46, -0.42)** |
| 19 | -1.97 | (-4.02, 0.09) | -1.19 | (-2.80, 0.42) |
| 20 | **-2.81** | **(-4.77, -0.85)** | -0.34 | (-1.63, 0.95) |
| 21 | **-4.44** | **(-7.36, -1.51)** | 0.56 | (-1.19, 2.31) |
| **Covariates** | | | | |
| Unemployment | 4.73 | (-2.05, 11.51) | 4.06 | (-2.43, 10.55) |
| Female | -7.35 | (-15.76, 1.06) | **-9.83** | **(-18.15, -1.51)** |
| White | -2.07 | (-9.15, 5.01) | -0.02 | (-6.47, 6.42) |
| Poverty | -**7.84** | **(-13.96, -1.72)** | **-7.34** | **(-13.18, -1.50)** |
| Data collection during COVID-19 | -1.49 | (-8.23, 5.25) | -0.73 | (-6.35, 4.89) |

1. CI: Confidence Interval
2. Significant results at P-value = 0.05 were in bold text

**Supplemental Table 2**: The estimates (95%CIs) from the time-varying effect model for negative affect score in the fall and winter months using binary food insecurity status ^1,2^

|  | **Fall** | | **Winter** | |
| --- | --- | --- | --- | --- |
|  | **b** | **95% CI** | **b** | **95% CI** |
| **Food insecurity status per study day** | | | | |
| 1 | -0.51 | (-2.79, 1.77) | 0.56 | (-1.61, 2.72) |
| 2 | -0.47 | (-2.55, 1.60) | -0.47 | (-2.14, 1.20) |
| 3 | -0.42 | (-2.36, 1.52) | -0.86 | (-2.36, 0.63) |
| 4 | -0.34 | (-1.94, 1.24) | -0.78 | (-2.13, 0.58) |
| 5 | -0.26 | (-1.36, 0.85) | -0.35 | (-1.57, 0.87) |
| 6 | -0.15 | (-0.92, 0.63) | 0.27 | (-0.89, 1.43) |
| 7 | -0.02 | (-0.91, 0.88) | 0.95 | (-0.21, 2.10) |
| 8 | 0.14 | (-1.00, 1.28) | **1.53** | **(0.42, 2.64)** |
| 9 | 0.31 | (-0.93, 1.54) | **1.95** | **(0.92, 2.97)** |
| 10 | 0.48 | (-0.79, 1.75) | **2.21** | **(1.09, 3.34)** |
| 11 | 0.65 | (-0.73, 2.02) | **2.35** | **(0.88, 3.82)** |
| 12 | 0.78 | (-0.77, 2.34) | **2.39** | **(0.51, 4.28)** |
| 13 | 0.88 | (-0.86, 2.62) | **2.37** | **(0.18, 4.55)** |
| 14 | 0.93 | (-0.86, 2.72) | **2.30** | **(0.06, 4.53)** |
| 15 | 0.91 | (-0.69, 2.51) | **2.21** | **(0.23, 4.18)** |
| 16 | 0.83 | (-0.40, 2.06) | **2.07** | **(0.44, 3.71)** |
| 17 | 0.73 | (-0.13, 1.59) | **1.85** | **(0.28, 3.42)** |
| 18 | 0.63 | (-0.11, 1.37) | 1.49 | (-0.34, 3.32) |
| 19 | 0.56 | (-0.49, 1.61) | 0.94 | (-1.13, 3.01) |
| 20 | 0.54 | (-1.27, 2.35) | 0.15 | (-1.99, 2.28) |
| 21 | 0.61 | (-2.58, 3.80) | -0.94 | (-3.41, 1.54) |
| **Covariates** | | | | |
| Unemployment | **8.59** | **(2.24, 14.94)** | **5.64** | **(1.04, 10.25)** |
| Female | 0.06 | (-8.23, 8.35) | 6.86 | (-0.38, 14.09) |
| White | 1.26 | (-3.60, 6.12) | -0.07 | (-4.32, 4.19) |
| Poverty | 0.52 | (-4.14, 5.18) | 0.93 | (-3.42, 5.27) |
| Data collection during COVID-19 | 1.25 | (-3.92, 6.42) | -1.06 | (-5.22, 3.11) |

1. CI: Confidence Interval
2. Significant results at P-value = 0.05 were in bold text.

**Supplemental Table 3.** The estimates (95%CIs) from the time-varying effect model for positive affect score pre- and during COVID-19 pandemic using binary food insecurity status ^1,2^

|  | **Pre-COVID-19 pandemic** | | **During COVID-19 pandemic** | |
| --- | --- | --- | --- | --- |
|  | **b** | **95% CI** | **b** | **95% CI** |
| **Food insecurity status per study day** | | | | |
| 1 | 1.75 | (-2.22, 5.72) | 0.55 | (-1.21, 2.31) |
| 2 | 1.47 | (-0.89, 3.84) | -1.18 | (-2.75, 0.38) |
| 3 | 1.24 | (-0.61, 3.08) | **-1.75** | **(-3.36, -0.15)** |
| 4 | 1.01 | (-0.49, 2.51) | **-1.48** | **(-2.96, -0.01)** |
| 5 | 0.76 | (-0.24, 1.77) | -0.68 | (-1.92, 0.57) |
| 6 | 0.47 | (-0.19, 1.12) | 0.33 | (-0.76, 1.42) |
| 7 | 0.09 | (-0.83, 1.01) | **1.23** | **(0.16, 2.29)** |
| 8 | -0.40 | (-1.59, 0.79) | **1.69** | **(0.69, 2.68)** |
| 9 | -0.99 | (-2.14, 0.16) | **1.58** | **(0.75, 2.41)** |
| 10 | **-1.61** | **(-2.64, -0.58)** | **1.03** | **(0.15, 1.91)** |
| 11 | **-2.19** | **(-3.35, -1.03)** | 0.19 | (-1.06, 1.44) |
| 12 | **-2.66** | **(-4.24, -1.08)** | -0.79 | (-2.43, 0.85) |
| 13 | **-2.94** | **(-4.94, -0.93)** | -1.75 | (-3.57, 0.07) |
| 14 | **-2.96** | **(-5.17, -0.74)** | **-2.55** | **(-4.19, -0.90)** |
| 15 | **-2.65** | **(-4.73, -0.58)** | **-3.03** | **(-4.21, -1.86)** |
| 16 | **-2.13** | **(-3.82, -0.44)** | **-3.19** | **(-4.54, -1.84)** |
| 17 | **-1.59** | **(-2.91, -0.26)** | **-3.09** | **(-5.36, -0.82)** |
| 18 | **-1.23** | **(-2.39, -0.07)** | -2.79 | (-5.82, 0.24) |
| 19 | **-1.26** | **(-2.43, -0.09)** | -2.37 | (-5.51, 0.78) |
| 20 | **-1.88** | **(-3.50, -0.26)** | -1.87 | (-4.40, 0.66) |
| 21 | **-3.30** | **(-6.41, -0.20)** | -1.38 | (-4.33, 1.56) |
| **Covariates** | | | | |
| Unemployment | **7.76** | **(2.06, 13.46)** | 2.07 | (-4.38, 8.52) |
| Female | -5.91 | (-12.87, 1.05) | -4.44 | (-14.44, 5.56) |
| White | **6.25** | **(0.64, 11.86)** | **-9.06** | **(-16.70, -1.42)** |
| Poverty | -4.44 | (-9.14, 0.26) | **-10.41** | **(-17.39, -3.43)** |
| Data collection in winter season | 1.13 | (-2.77, 5.03) | 1.18 | (-5.01, 7.37) |

1. CI: Confidence Interval
2. Significant results at P-value = 0.05 were in bold text.

**Supplemental Table 4.** The estimates (95%CIs) from the time-varying effect model for negative affect score pre- and during COVID-19 pandemic using binary food insecurity status ^1,2^

|  | **Pre-COVID-19 pandemic** | | **During COVID-19 pandemic** | |
| --- | --- | --- | --- | --- |
|  | **b** | **95% CI** | **b** | **95% CI** |
| **Food insecurity status per study day** | | | | |
| 1 | 1.76 | (0.13, 3.38) | **-1.80** | **(-3.45, -0.14)** |
| 2 | 0.96 | (-0.83, 2.73) | **-1.86** | **(-3.17, -0.54)** |
| 3 | 0.42 | (-1.55, 2.39) | **-1.66** | **(-2.78, -0.53)** |
| 4 | 0.11 | (-1.63, 1.86) | **-1.27** | **(-2.28, -0.27)** |
| 5 | 0.01 | (-1.25, 1.27) | -0.79 | (-1.81, 0.23) |
| 6 | 0.09 | (-0.79, 0.97) | -0.28 | (-1.49, 0.93) |
| 7 | 0.31 | (-0.65, 1.27) | 0.18 | (-1.28, 1.63) |
| 8 | 0.66 | (-0.51, 1.83) | 0.51 | (-1.13, 2.14) |
| 9 | 1.09 | (-0.15, 2.33) | 0.68 | (-1.01, 2.36) |
| 10 | **1.54** | **(0.20, 2.88)** | 0.73 | (-0.92, 2.38) |
| 11 | **1.96** | **(0.32, 3.60)** | 0.72 | (-0.85, 2.30) |
| 12 | **2.29** | **(0.24, 4.35)** | 0.70 | (-0.81, 2.21) |
| 13 | **2.48** | **(0.08, 4.88)** | 0.71 | (-0.75, 2.18) |
| 14 | 2.45 | (-0.05, 4.95) | 0.82 | (-0.59, 2.23) |
| 15 | 2.18 | (-0.04, 4.40) | 1.05 | (-0.25, 2.36) |
| 16 | **1.69** | **(0.02, 3.37)** | **1.38** | **(0.21, 2.54)** |
| 17 | 1.11 | (-0.05, 2.27) | **1.70** | **(0.66, 2.74)** |
| 18 | 0.53 | (-0.53, 1.58) | **1.92** | **(0.97, 2.87)** |
| 19 | 0.06 | (-1.29, 1.40) | **1.94** | **(1.01, 2.86)** |
| 20 | -0.19 | (-2.04, 1.66) | **1.67** | **(0.52, 2.81)** |
| 21 | -0.12 | (-3.04, 2.80) | 1.00 | (-0.86, 2.87) |
| **Covariates** | | | | |
| Unemployment | **9.77** | **(3.91, 16.63)** | 4.19 | (-1.08, 9.46) |
| Female | 1.86 | (-4.98, 8.70) | **12.06** | **(4.04, 20.08)** |
| White | 1.30 | (-3.11, 5.71) | 0.76 | (-4.22, 5.74) |
| Poverty | 1.22 | (-3.37, 5.81) | -2.43 | (-7.13, 2.27) |
| Data collection in winter season | -0.72 | (-5.03, 3.59) | -1.04 | (-5.88, 3.80) |

1. CI: Confidence Interval
2. Significant results at P-value = 0.05 were in bold text.

**Supplemental Table 5.** The estimates (95%CIs) from the time-varying effect model for positive affect score in the fall and winter months using continuous food insecurity scores^1,2^

|  | **Fall** | | **Winter** | |
| --- | --- | --- | --- | --- |
|  | **b** | **95% CI** | **b** | **95% CI** |
| **Total food insecurity scores per study day** | | | | |
| 1 | 0.96 | (-1.48, 3.40) | 1.67 | (-1.78, 5.13) |
| 2 | -0.16 | (-2.35, 2.04) | 0.70 | (-1.35, 2.75) |
| 3 | -0.59 | (-2.84, 1.65) | 0.21 | (-1.12, 1.54) |
| 4 | -0.55 | (-2.47, 1.37) | 0.05 | (-0.85, 0.96) |
| 5 | -0.21 | (-1.49, 1.07) | 0.10 | (-0.52, 0.72) |
| 6 | 0.23 | (-0.52, 0.98) | 0.21 | (-0.46, 0.89) |
| 7 | 0.58 | (-0.39, 1.56) | 0.26 | (-0.64, 1.17) |
| 8 | 0.66 | (-0.68, 2.00) | 0.11 | (-0.86, 1.09) |
| 9 | 0.37 | (-1.06, 1.80) | -0.28 | (-1.08, 0.51) |
| 10 | -0.16 | (-1.55, 1.24) | **-0.86** | **(-1.52, -0.19)** |
| 11 | -0.82 | (-2.28, 0.64) | **-1.51** | **(-2.46, -0.56)** |
| 12 | -1.49 | (-3.19, 0.21) | **-2.15** | **(-3.57, -0.73)** |
| 13 | **-2.05** | **(-4.01, -0.08)** | **-2.70** | **(-4.47, -0.93)** |
| 14 | **-2.37** | **(-4.44, -0.31)** | **-3.06** | **(-4.90, -1.22)** |
| 15 | **-2.36** | **(-4.26, -0.46)** | **-3.15** | **(-4.70, -1.60)** |
| 16 | **-2.11** | **(-3.81, -0.41)** | **-2.96** | **(-4.13, -1.80)** |
| 17 | **-1.81** | **(-3.56, -0.05)** | **-2.55** | **(-3.74, -1.35)** |
| 18 | -1.68 | (-3.61, 0.26) | **-1.94** | **(-3.46, -0.42)** |
| 19 | -1.93 | (-3.87, 0.01) | -1.19 | (-2.80, 0.42) |
| 20 | **-2.77** | **(-4.62, -0.91)** | -0.34 | (-1.63, 0.95) |
| 21 | **-4.41** | **(-7.29, -1.53)** | 0.56 | (-1.19, 2.31) |
| **Covariates** | | | | |
| Unemployment | 4.45 | (-2.42, 11.33) | 4.06 | (-2.43, 10.55) |
| Female | -7.24 | (-15.50, 1.02) | **-9.83** | **(-18.15, -1.51)** |
| White | -1.91 | (-9.07, 5.25) | -0.02 | (-6.47, 6.42) |
| Poverty | -**7.60** | **(-13.78, -1.42)** | **-7.34** | **(-13.18, -1.50)** |
| Data collection during COVID-19 | -1.53 | (-8.29, 5.22) | -0.73 | (-6.35, 4.89) |

1. CI: Confidence Interval
2. Significant results at P-value = 0.05 were in bold text

**Supplemental Table 6**: The estimates (95%CIs) from the time-varying effect model for negative affect score in the fall and winter months using continuous food insecurity scores ^1,2^

|  | **Fall** | | **Winter** | |
| --- | --- | --- | --- | --- |
|  | **b** | **95% CI** | **b** | **95% CI** |
| **Total food insecurity scores per study day** | | | | |
| 1 | -0.52 | (-2.77, 1.73) | 0.56 | (-1.61, 2.72) |
| 2 | 0.30 | (-2.43, 1.83) | -0.47 | (-2.14, 1.20) |
| 3 | 0.17 | (-2.20, 1.87) | -0.86 | (-2.36, 0.63) |
| 4 | 0.08 | (-1.97, 1.62) | -0.78 | (-2.13, 0.58) |
| 5 | 0.04 | (-1.24, 1.17) | -0.35 | (-1.57, 0.87) |
| 6 | 0.01 | (-0.81, 0.82) | 0.27 | (-0.89, 1.43) |
| 7 | 0.06 | (-0.80, 0.92) | 0.95 | (-0.21, 2.10) |
| 8 | 0.16 | (-0.92, 1.24) | **1.53** | **(0.42, 2.64)** |
| 9 | 0.31 | (-0.87, 1.49) | **1.95** | **(0.92, 2.97)** |
| 10 | 0.48 | (-0.75, 1.71) | **2.21** | **(1.09, 3.34)** |
| 11 | 0.66 | (-0.69, 2.01) | **2.35** | **(0.88, 3.82)** |
| 12 | 0.82 | (-0.73, 2.36) | **2.39** | **(0.51, 4.28)** |
| 13 | 0.93 | (-0.80, 2.67) | **2.37** | **(0.18, 4.55)** |
| 14 | 0.99 | (-0.80, 2.78) | **2.30** | **(0.06, 4.53)** |
| 15 | 0.96 | (-0.64, 2.56) | **2.21** | **(0.23, 4.18)** |
| 16 | 0.86 | (-0.36, 2.08) | **2.07** | **(0.44, 3.71)** |
| 17 | 0.73 | (-0.10, 1.57) | **1.85** | **(0.28, 3.42)** |
| 18 | 0.61 | (-0.07, 1.29) | 1.49 | (-0.34, 3.32) |
| 19 | 0.52 | (-0.46, 1.51) | 0.94 | (-1.13, 3.01) |
| 20 | 0.51 | (-1.25, 2.28) | 0.15 | (-1.99, 2.28) |
| 21 | 0.62 | (-2.54, 3.79) | -0.94 | (-3.41, 1.54) |
| **Covariates** | | | | |
| Unemployment | **8.40** | **(2.06, 14.73)** | **5.64** | **(1.04, 10.25)** |
| Female | 0.14 | (-8.21, 8.48) | 6.86 | (-0.38, 14.09) |
| White | 1.37 | (-3.45, 6.19) | -0.07 | (-4.32, 4.19) |
| Poverty | 0.64 | (-4.02, 5.30) | 0.93 | (-3.42, 5.27) |
| Data collection during COVID-19 | 1.22 | (-3.94, 6.38) | -1.06 | (-5.22, 3.11) |

1. CI: Confidence Interval
2. Significant results at P-value = 0.05 were in bold text.

**Supplemental Figures**


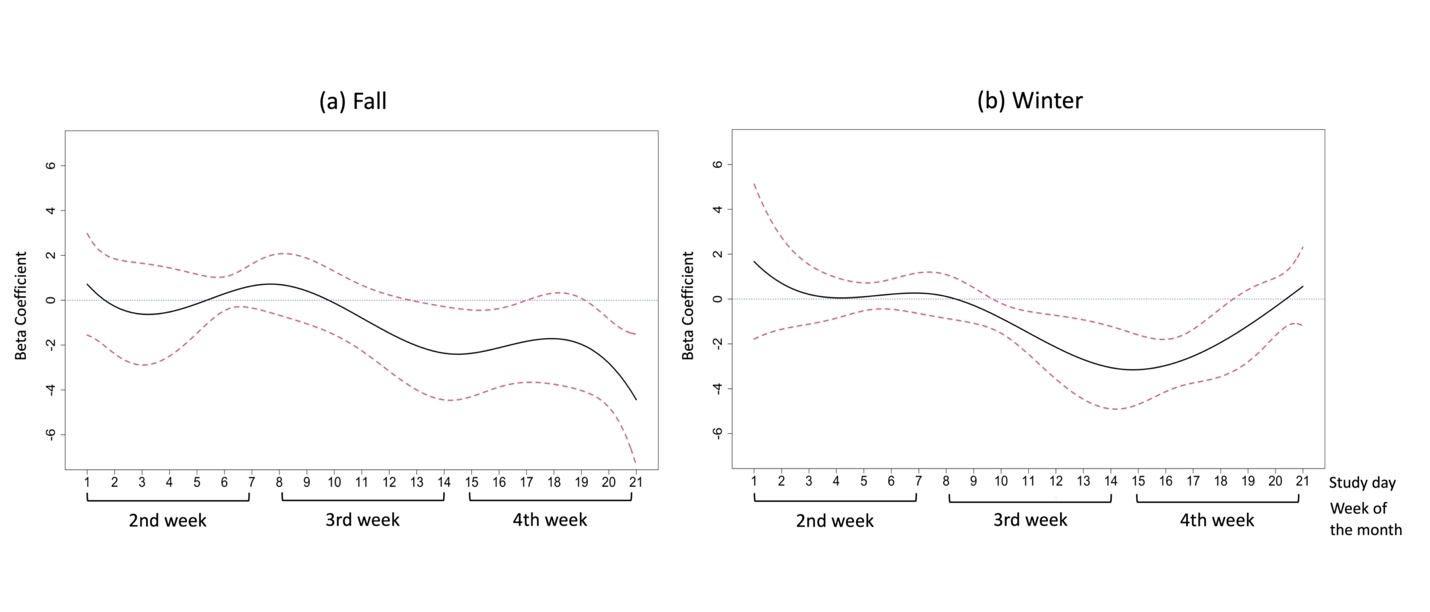


**Supplemental Figure 1.** The association between daily food insecurity scores and positive affect in (a) the fall months; (b) the winter months. The time-varying effect models were adjusted for gender, race/ethnicity, employment, poverty status, and data collection pre- or during covid-19. The solid line in black represents the estimated coefficients between daily food insecurity scores and positive affect. The dashed lines in red are the pointwise 95% confidence intervals. The dotted blue line at zero represents null association between daily food insecurity scores and affective well-being.


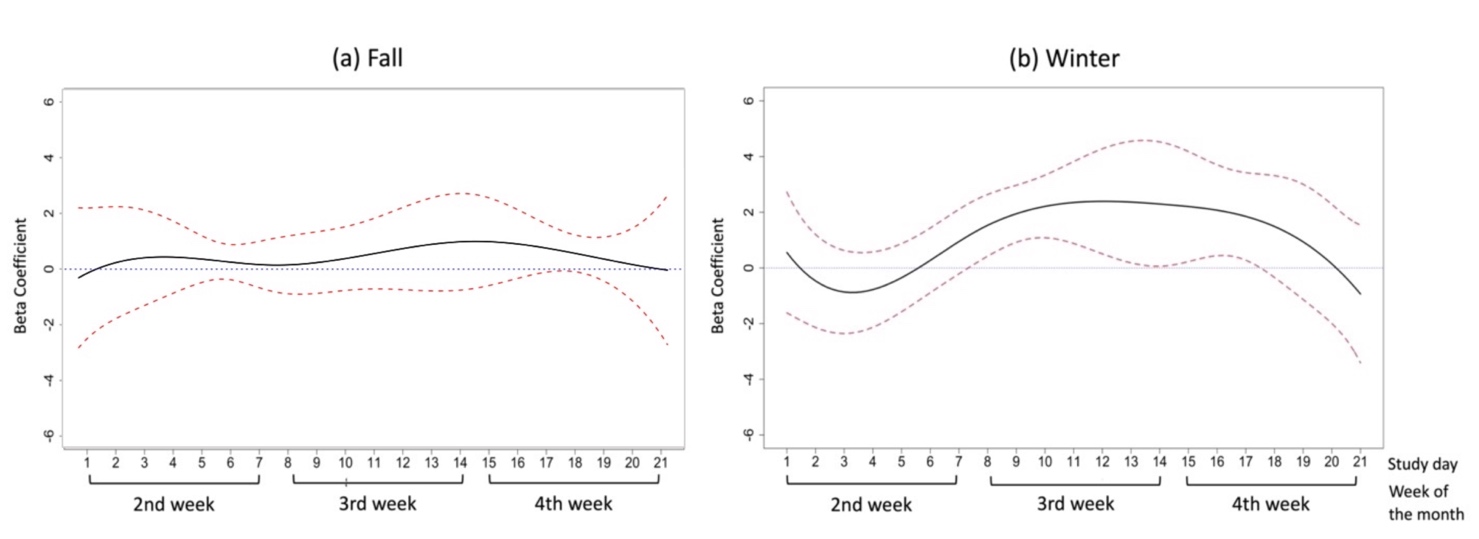


**Supplemental Figure 2.** The association between daily food insecurity scores and negative affect in (a) fall months; (b) winter months. The time-varying effect models were adjusted for gender, race/ethnicity, employment, poverty status, and data collection pre- or during covid-19. The solid line in black represents the estimated coefficients between daily food insecurity scores and negative affect. The dashed lines in red are the pointwise 95% confidence intervals. The dotted blue line at zero represents null association between daily food insecurity scores and affective well-being.
